# Potential Molecular Targets of Tenofovir Disoproxil Fumarate for Alleviating Chronic Liver Diseases *via* a Non-Antiviral Effect in a Normal Mouse Model

**DOI:** 10.3389/fmolb.2021.763150

**Published:** 2021-11-16

**Authors:** Yuanqin Duan, Zhiwei Chen, Hu Li, Wei Shen, Yi Zeng, Mingli Peng, Peng Hu

**Affiliations:** Key Laboratory of Molecular Biology for Infectious Diseases (Ministry of Education), Department of Infectious Diseases, Institute for Viral Hepatitis, The Second Affiliated Hospital of Chongqing Medical University, Chongqing, China

**Keywords:** tenofovir disoproxil fumarate, chronic liver diseases, non-antiviral effect, immunity, inflammation, metabolism, miR-155-5p, NF-κB

## Abstract

Accumulating evidence suggests that tenofovir disoproxil fumarate (TDF) can attenuate liver fibrosis directly, the mechanism of which, however, has not been fully elucidated, and there is a paucity of data concerning whether TDF can also mitigate other chronic liver diseases (CLDs). We aimed to identify the molecular targets and potential mechanism of TDF itself in ameliorating CLDs. RNA-sequencing was performed on mouse liver tissues treated with TDF or normal saline. Then the differentially expressed genes (DEGs) were screened, and enrichment analyses of the function and signaling pathways of DEGs were performed with Database for Annotation, Visualization, and Integrated Discovery (DAVID) and Metascape. Next, protein-protein interaction (PPI) networks were constructed and module analyses were utilized to identify significant genes. Subsequently, the DisGeNET platform was used to identify the potential target genes of TDF in mitigating these diseases. Finally, prediction of the transcription factors (TFs) and microRNAs (miRNAs) of the target genes was done to conjecture the underlying mechanism by which TDF relieved CLDs. As a result, a total of 854 DEGs were identified, and the DEGs were involved mainly in “immunity,” “inflammation,” and “metabolism” processes. In addition, 50 significant genes were obtained *via* PPI construction and module analyses. Furthermore, by means of DisGeNET, 19 genes (*Adra2a, Cxcl1, Itgam, Cxcl2, Ccr1, Ccl5, Cxcl5, Fabp5, Sell, Lilr4b, Ccr2, Tlr2, Lilrb4a, Tnf, Itgb2, Lgals3, Cxcr4, Sucnr1,* and *Mme*) were identified to be associated with nine CLDs. Finally, 34 miRNAs (especially mmu-miR-155-5p) and 12 TFs (especially Nfkb1) were predicted to be upstream of the nine target genes (*Cxcl1, Cxcl2, Ccl5, Ccr2, Sell, Tlr2, Tnf, Cxcr4,* and *Mme*) of TDF in ameliorating CLDs. In conclusion, our study suggests that TDF have the potential to ameliorate CLDs independently of its antiviral activity by affecting the expression of genes involved in hepatic immune, inflammatory, and metabolic processes *via* mmu-miR-155-5p-NF-κB signaling. These findings provided *prima facie* evidence for using TDF in CHB patients with concurrent CLDs.

## Introduction

Tenofovir disoproxil fumarate (TDF), an orally administered ester prodrug of tenofovir, is widely used for effective treatment of hepatitis B virus (HBV) infection (Perry and Simpson, 2009). The REVEAL-HBV study group reported an increased serum level of HBV DNA at baseline to be a strong and independent risk predictor of chronic liver diseases (CLDs) development (Chen, 2006; Iloeje et al., 2006). Numerous studies have shown that TDF can achieve sustained suppression of HBV in the long-term management of chronic hepatitis B (CHB) patients regardless of hepatitis B e antigen’s status and ethnicity (Heathcote et al., 2011; Gordon et al., 2013; Marcellin et al., 2019). Meanwhile, long-term studies have demonstrated sustained suppression of HBV replication with TDF to be associated with regression and a reduced risk of CLDs in CHB patients (Marcellin et al., 2013; Liu et al., 2019). Those effects were considered to be due mainly to reduced hepatic damage caused by HBV infection, but the direct non-antiviral effects of TDF might also be involved.

Two abstracts demonstrated that TDF could regress liver fibrosis directly by blocking proliferation (Signal Transduction and Cell Function, 2013) and inducing apoptosis of activated hepatic stellate cells (Abstracts, 2020). Recently, a basic study showed that TDF could attenuate liver fibrosis by upregulating expression of hepatitis C virus’s non-structural protein 5A transactivated protein 9 (NS5ATP9), thereby inhibiting TGFβ1/Smad3 and NF-κB/NLRP3 signaling pathways (Zhao et al., 2020). However, the mechanism by which TDF mitigates liver fibrosis has not been elucidated fully. Furthermore, there are no data suggesting whether TDF can also alleviate other CLDs independently of its antiviral activity.

We wished to explore the potential mechanism and molecular targets of TDF in improving CLDs. Hence, we undertook RNA-sequencing (RNA-seq) on the liver tissues of wild-type mice treated with TDF and employed an integrated bioinformatic analysis.

## Materials and Methods

### TDF

TDF was kindly gifted by Guangshengtang Co., Ltd (Fujian, China). The purity of TDF was 99.5%.

### Animals and TDF Treatment

**FIGURE 1:**
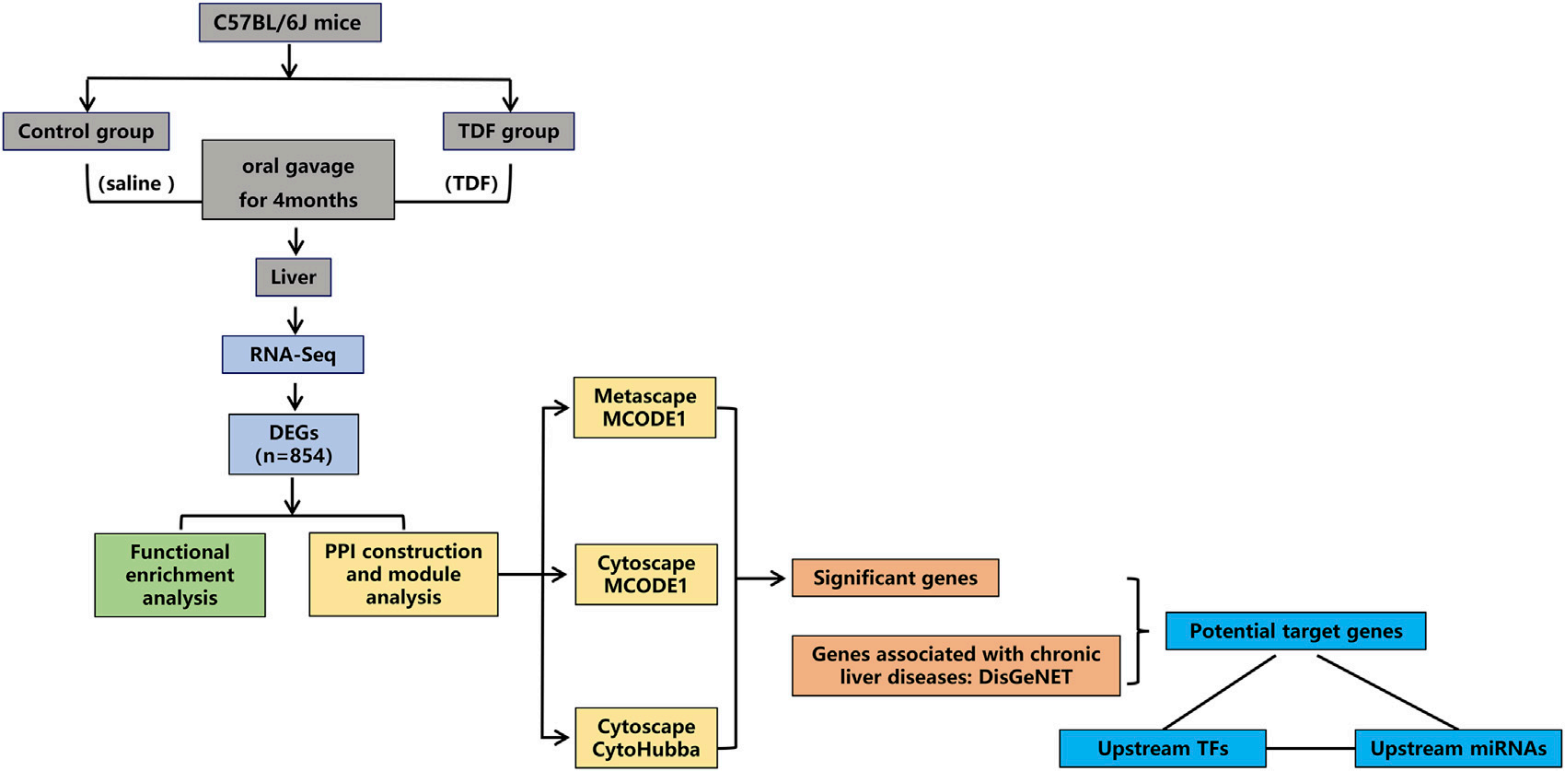
Flowchart of this research.

The study protocol was approved by the Animal Protection Organization and Ethics Committee of Chongqing Medical University (Chongqing, China). Female C57BL/6J mice (8 weeks) from the Animal Center of Chongqing Medical University were housed in a room with a 12-h light and dark cycle at 22°C with free access to mouse chow and water. After 1 week of acclimatization, mice were divided randomly into two groups. Mice in the TDF group were administered with TDF solution (455 mg of TDF powder + 0.5 g of sodium carboxymethyl cellulose +100 ml of normal saline were mixed thoroughly with a homogenizer until the solution was transparent) at 45.5 mg/kg/day by oral gavage for 4 months. Dose determination of TDF was performed *via* conversion of human equivalent doses to murine doses based on the body surface area (Reagan‐Shaw et al., 2008; Ng et al., 2015). Mice in the control group received an equivalent volume of vehicle (100 ml of normal saline + 0.5 g of sodium carboxymethyl cellulose were mixed thoroughly with a homogenizer until the solution was transparent) for 4 months. Each mouse was weighed once a week. At study termination, mice were killed after 12 h of fasting. Blood samples were taken by excising the eyeballs. The liver was collected and weighed for subsequent experiments. A flowchart of this study is shown in Figure 1.

### Biochemical Parameters

Serum was collected after centrifugation at 1,000 rpm for 15 min. Serum ALT levels were measured in the Clinical Laboratory, The Second Affiliated Hospital, Chongqing Medical University.

### Hematoxylin and Eosin Staining

Liver tissues of mice were fixed in 4% paraformaldehyde, embedded in paraffin, sliced, and stained with hematoxylin and eosin (H&E).

### Isolation and Sequencing of RNA

According to manufacturer instructions, TRIzol^®^ (Invitrogen, United States) was used to extract total RNA from the liver. Then a bioanalyzer (2,200 series; Agilent Technologies, United States) was used to evaluate the concentration, purity, and quality of RNA. Then RNA was sequenced using the DNBseq platform in BGI (Shenzhen, China).

### Differential Gene Expression

Low-quality reads, adaptor reads, and reads with > 10% unknown bases (poly-N) were removed from raw data to obtain high-quality “clean” reads for subsequent analyses. The Q20 (percentage of bases with a quality value ≥ 20) and Q30 content of clean data were also calculated. Clean reads were mapped to the *Mus musculus* reference genome (GRCm38.p6) using HISAT2 (http://daehwankimlab.github.io/hisat2/) (Kim et al., 2015). Expression was calculated by RSEM (Li and Dewey, 2011) and represented in fragments per kilobase per million (FPKM) reads. Differential gene expression was identified using the “DEGseq” package with R (R Institute for Statistical Computing, Vienna, Austria) (Wang et al., 2010). The absolute value of fold change (FC) ≥ 2 and adjusted *p*-value (Q-value) ≤ 0.001 were adopted as criteria for determining the significance of differential expression of a particular gene.

### Functional Enrichment Analyses of DEGs *via* DAVID and Metascape

The Gene Ontology (GO) database and Kyoto Encyclopedia of Genes and Genomes (KEGG) database were employed to identical enrichment of function and signaling pathways, respectively, based on Database for Annotation, Visualization, and Integrated Discovery (DAVID, https://david.ncifcrf.gov/) (Huang et al., 2009). Following the instructions of the DAVID manual, first, we clicked on the “Start Analysis” on the Internet website. Second, we entered the DEGs list, selected identifiers as “entrez gene ID,” selected list types as “gene lists,” and submitted lists. Third, we chose to limit annotations and background by *M. musculus*. Finally, the enrichment results of GO and KEGG databases were presented. *p* < 0.05 and gene count ≥ 2 were considered significant.

Furthermore, additional analyses of enrichment of function signaling pathways were done using Metascape (https://metascape.org/gp/index.html#/main/step1/) (Zhou et al., 2019). First, we pasted the gene list as “entrez gene ID.” Second, we chose to input the species as *M. musculus*. Third, we clicked on “Express Analysis.” Finally, analyses of enrichment of function and signaling pathways were carried out with the following ontology sources: Biological Process (BP) within the GO database, KEGG Pathway, Reactome Gene Sets, CORUM, TRRUST, PaGenBase, WikiPathways, and PANTHER Pathway. Terms with *p* < 0.01, minimum count of 3, and enrichment factor >1.5 (the enrichment factor is the ratio between the observed counts and the counts expected by chance) were collected and grouped into clusters based on their membership similarities.

### Construction of Protein-Protein Interaction (PPI) Networks, Significant Modules, and a “Hub Gene” Network

First, Metascape was utilized to construct a PPI network and identify the significant modules. Besides, the Search Tool for the Retrieval of Interacting Genes (STRING, https://string-db.org/), a user-friendly online system that provides predicted and experimental interactions of proteins (Szklarczyk et al., 2019), was used to establish a PPI network of DEGs with a confidence score ≥ 0.7 for significant differences. Then the PPI network was visualized using Cytoscape 3.6.1 (www.cytoscape.org) (Shannon, 2003). Molecular Complex Detection (MCODE) 1.5.1 (a plugin of Cytoscape) (Bader and Hogue, 2003) was used to screen and identify the most significant modules in the PPI network. MCODEs were extracted when the node score cutoff was 0.2 and K-core was 2. cytoHubba (a plugin of Cytoscape) was employed to calculate the properties of the network topology for nodes to identify hub genes with a degree ≥ 10. The “degree” indicates the number of edges connected with a specific node. Nodes with a high degree are identified as hub genes (i.e., may contribute to vital biological behaviors).

### Identification and Analyses of Significant Genes

A Venn diagram was delineated to identify significant union genes among “Metascape_MCODE,” “Cytoscape_MCODE,” and “Cytoscape_cytoHubba” by Bioinformatics (www.bioinformatics.com.cn/), an online platform for the analyses and visualization of data. Summaries for the basic information of the significant genes were obtained *via* Mouse Genome Informatics (www.informatics.jax.org/). A hierarchical clustering heatmap of significant genes was plotted by using OriginPro 2021 based on gene expression, and classified by the biological function of genes. Correlation analyses among significant genes were achieved through Pearson’s correlation test.

### Identification of Potential Target Genes of TDF for Ameliorating CLDs

DisGeNET 7.0 (www.disgenet.org/) is a discovery platform containing one of the largest publicly available collections of genes and variants associated to human diseases (Piñero et al., 2019). The current version of DisGeNET contains 1,134,942 gene–disease associations (GDAs), between 21,671 genes and 30,170 diseases, disorders, traits, and clinical or abnormal human phenotypes. The relationships between the significant genes and nine common CLDs were analyzed *via* this tool. First, we entered the name of the diseases in the “Search” box. Subsequently, the summary of the GDA score and evidence for GDAs were presented, and the results were downloaded. Finally, the target genes associated with CLDs were identified from the results.

### Prediction of Transcription Factors (TFs) and MicroRNAs (miRNAs) and Construction of TF-miRNA Co-regulatory Networks of Target Genes

To further explore how TDF improves CLDs, Transcriptional Regulatory Relationships Unraveled by Sentence-based Text mining (TRRUST 2, https://www.grnpedia.org/trrust/), a database containing 6552 TF–target interactions for 828 mouse TFs (Han et al., 2018), was employed to predict the TFs of target genes. First, we selected the species as “mouse,” and submitted the list of target genes in the bottom panel of the search area titled “Find key regulators for query genes.” Then the results were downloaded. Meanwhile, prediction of miRNAs of the target genes and TFs identified from TRRUST was done using DIANA-TarBase v8 (www.microrna.gr/tarbase/), a database that curates experimentally verified miRNA targets manually and contains 665,843 unique miRNA–target pairs (Karagkouni et al., 2018). We defined the species as *M. musculus*, entered the genes and TFs one-by-one, and tabulated the results. Ultimately, pairwise-related genes, TFs, and miRNAs were screened, and Cytoscape was utilized to visualize the TF–miRNA co-regulatory networks of the target genes.

## Results

### TDF had No Significant Effect on Liver Function of Normal Mice

TDF was administered to mice by oral gavage for 4 months to simulate the long-term use of TDF in humans. Before intervention, there was no significant difference in the body weight (BW) of mice (Supplementary Figure S1A) between the control group and TDF group. At study termination, no mice died from TDF administration. Besides, no significant differences were found between the TDF group (n = 9) and control group (n = 11) in BW (Supplementary Figure S1B), liver weight (LW) (Supplementary Figure S1C), liver index (LW/BW) (Supplementary Figure S1D), ALT level (Supplementary Figure S1E), and liver histology (Supplementary Figure S1F). Taken together, these results indicated that the TDF dose we employed was non-toxic, and had no significant effect on liver function of normal mice.

### RNA-Seq and Read Mapping

To explore the transcriptional changes in the liver induced by TDF administration, RNA-seq was done using the liver tissues of the mice: 639.99 million raw reads (Supplementary Table S1) were generated. After removing adaptors and low-quality reads, we obtained 616.74 million clean reads, with a high quality of Q30 ≥ 93.36%. Then the trimmed clean reads were mapped onto the *M. musculus* reference genome, and 76.32–81.35% of clean reads were mapped uniquely to the genome (Supplementary Table S1B). The uniquely mapped reads were used in all subsequent analyses.

### A Total of 854 Annotated Genes Were Identified to be DEGs Induced by TDF in Mouse Livers

**FIGURE 2:**
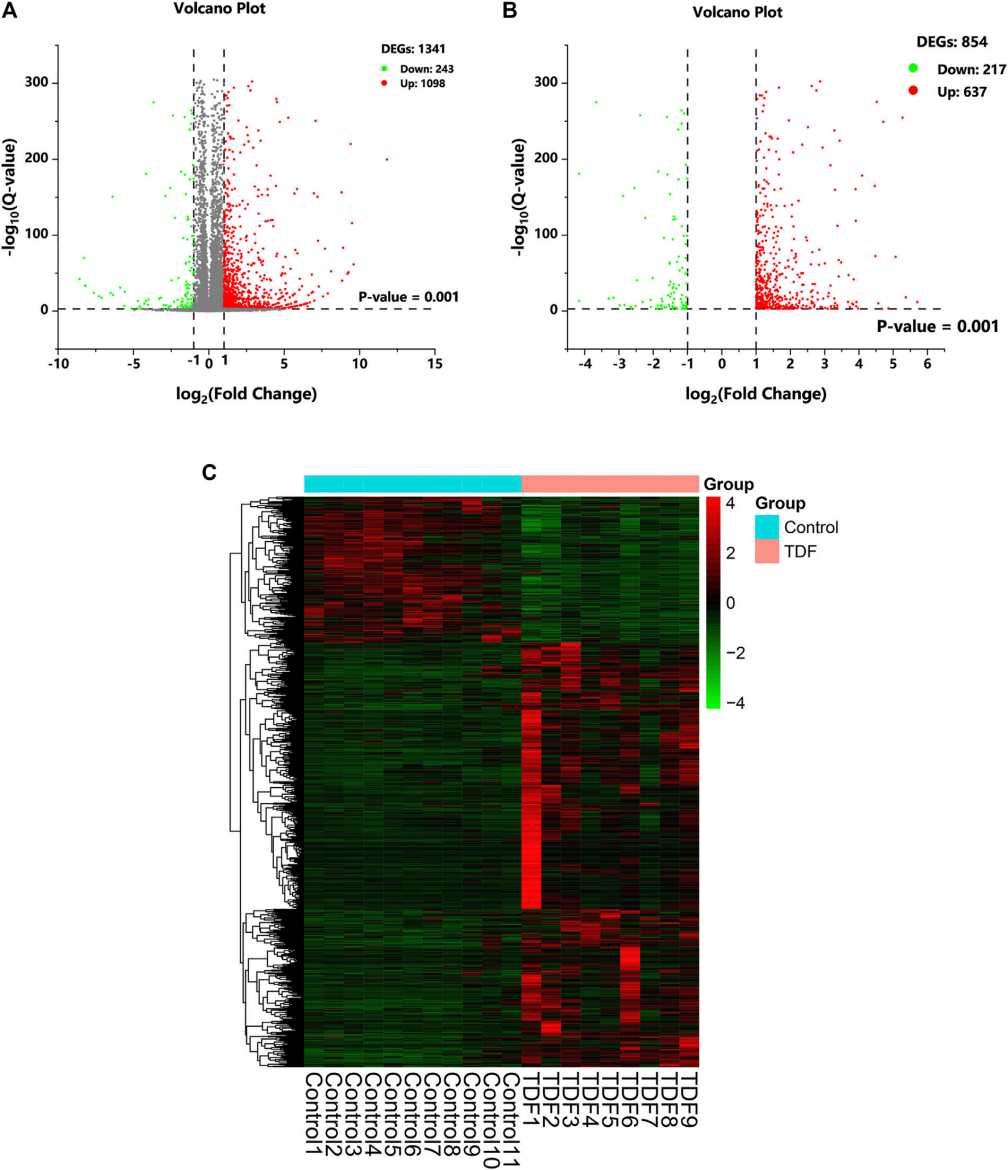
Differentially expressed genes (DEGs) in response to TDF. **(A, B)** The volcano plot of DEGs. The cutoff values fold change ≥ 2 and Q-value ≤ 0.001 were utilized to identify DEGs. Downregulated DEGs are marked in green, upregulated DEGs are marked in red, and unchanged genes are marked in gray. **(C)** Hierarchical clustering of DEGs.

A total of 17,199 mRNAs were annotated (Supplementary Table S2). DEGseq was employed to screen for DEGs. A total of 1,341 annotated genes were identified to be differentially expressed when considering exclusively a stringent threshold of Q-value ≤ 0.001 and an absolute value of FC ≥ 2, which is presented as a volcano plot (Figure 2A). After removing DEGs that caused differences between groups due to abnormal expression within the group, we obtained 854 DEGs eventually (217 downregulated DEGs and 637 upregulated DEGs) (Figure 2B and Supplementary Table S3). Hence, TDF could affect gene expression in mouse livers directly. To obtain a global view of these 854 DEGs, hierarchical clustering (Figure 2C) was done with normalized FPKM values, and indicated that our samples were of “good” quality with gene expression of similar proportion in each group.

### Functional Enrichment Analyses Indicated That the 854 DEGs Induced by TDF Were Involved Mainly in “Immunity,” “Inflammation,” and “Metabolism” Processes

**FIGURE 3:**
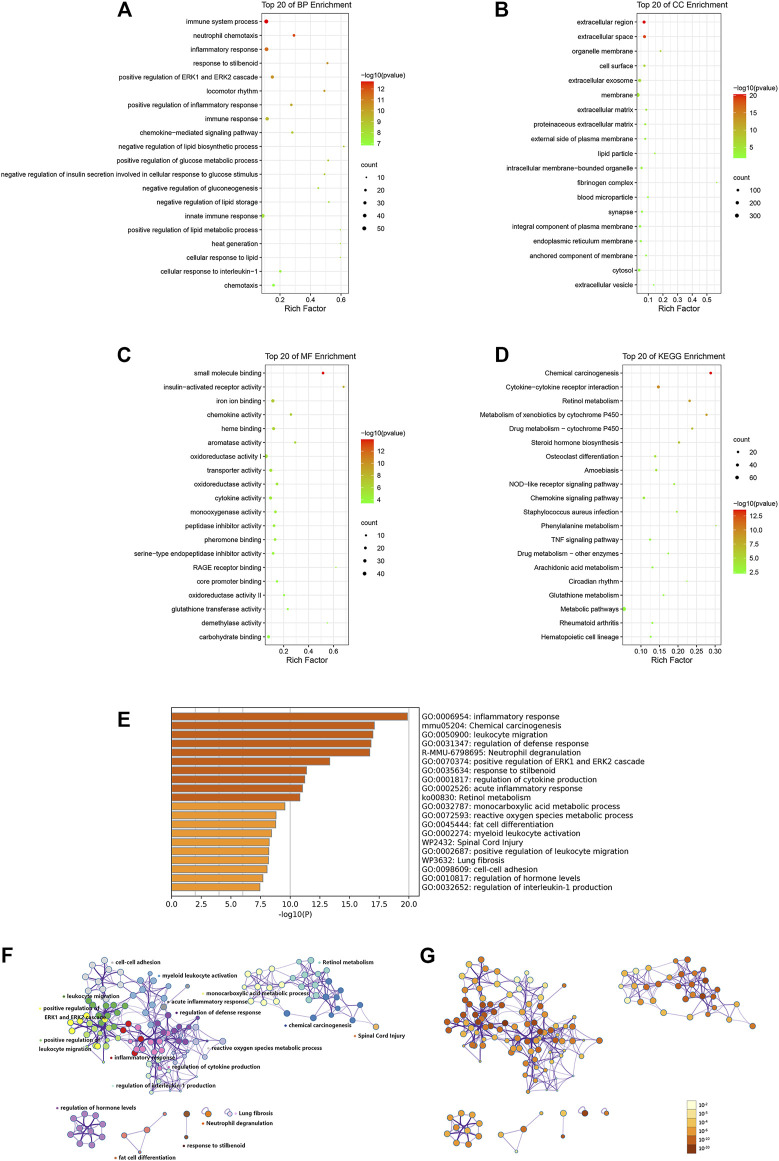
Functional enrichment analyses of DEGs *via* DAVID **(A–D)** and Metascape **(E–G)**. Variations in DEGs associated with **(A)** biological process, **(B)** cellular component, **(C)** molecular function, and **(D)** KEGG analysis. Rich factor is the ratio of the DEG number to the total gene number in a certain pathway. The color and size of the dots represent the range of the p-value and the number of DEGs mapped to the indicated pathways, respectively. **(E)** Bar graph of enriched clusters across inputted DEGs lists, colored by p-values. Network of enriched terms: **(F)** colored by cluster ID, where nodes that share the same cluster ID are typically close to each other, **(G)** colored by p-value, where terms containing more genes tend to have a more significant p-value. The top 20 significant enriched pathways are shown.

First, enrichment analyses using the GO database and KEGG database were undertaken using DAVID. We discovered that 774 out of 854 profiled DEGs were assigned to 394 GO terms: 264 for biological process (BP), 32 for cellular component (CC), and 98 for molecular function (MF). Variations in DEGs related to BP were involved mainly in “immune system process,” “inflammatory response,” “lipid metabolic process,” and “glucose metabolic process” (Figure 3A). With regard to CC, DEGs were significantly enriched in the “extracellular region,” “extracellular space,” “organelle membrane,” and “cell surface” (Figure 3B). Variations in DEGs associated with MF were significantly enriched in “small molecule binding,” “insulin-activated receptor activity,” “iron ion binding,” and “chemokine activity” (Figure 3C). The KEGG database indicated that 356 out of 854 profiled DEGs were assigned to 46 signaling pathways. The significant pathways relevant to DEGs were immune, inflammatory pathways (“cytokine-cytokine receptor interaction,” “NOD-like receptor signaling pathway,” “chemokine signaling pathway,” and “TNF signaling pathway”), and metabolic pathways (“retinol metabolism,” “steroid hormone biosynthesis,” “arachidonic acid metabolism,” and “glutathione metabolism”) (Figure 3D). The data of GO and KEGG classifications are shown in Supplementary Table S4.

To gain further insight into the functions of DEGs, analyses of enrichment of signaling pathways and function were carried out *via* Metascape. The results (in accordance with the results using DAVID) indicated that the DEGs induced by TDF were significantly enriched in inflammatory processes (“leukocyte migration,” “neutrophil degranulation,” “acute inflammatory response,” “positive regulation of leukocyte migration,” and “regulation of interleukin-1 production”) and metabolic processes (“retinol metabolism” and “monocarboxylic acid metabolism”) (*p* < 0.05, Figures 3E–G).

### PPI Construction and Module Analyses Identified 50 Genes as Significant Genes, and These Genes Were Involved Mainly in “Immunity,” “Inflammation,” and “Metabolic” Processes

**FIGURE 4:**
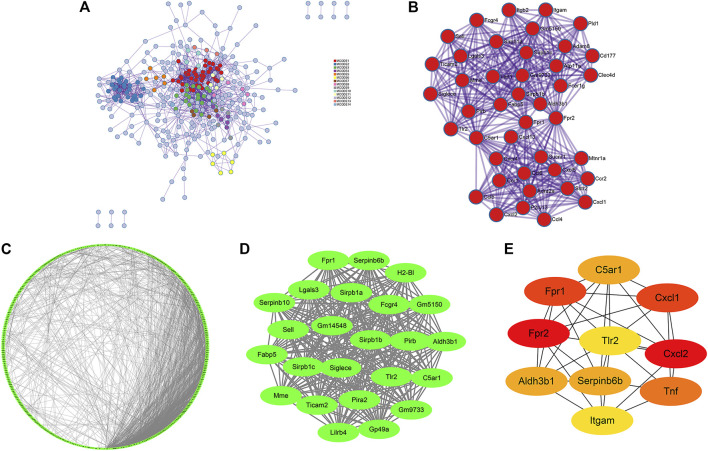
PPI network, significant modules and hub gene networks constructed through Metascape **(A, B)**, STRING **(C)**, and Cytoscape **(D, E)**. **(A)** A PPI network of DEGs including 14 MCODE modules was established *via* Metascape. **(B)** The Metascape_MCODE1 consists of 42 genes. **(C)** A PPI network of DEGs consisting of 1937 edges and 443 nodes was established *via* STRING with a confidence score of ≥ 0.7 for significant differences. **(D)** The Cytoscape_MCODE1 consists of 24 genes. **(E)** Ten genes were identified as hub genes with Cytoscape_cytoHubba.

First, a PPI network of DEGs was constructed through Metascape (Figure 4A). Fourteen MCODE modules were identified from the PPI network. Notably, MCODE1 with the highest score consisted of 42 genes: *Sirpb1c, Gm9733, Sirpb1b, Gm5150, Sirpb1a, Fcgr4, Ticam2, Sucnr1, Siglece, P2ry13, Cd177, Aldh3b1, Cxcl13, Lair1, Atp11a, Tlr2, Sstr2, Sell, Cxcl5, Cxcl2, Ccl6, Ccl5, Ccl4, Pld1, Pirb, Mtnr1a, Clec4d, Mme, Lgals3, Fabp5, Itgb2, Itgam, Cxcl1, Fpr1, Fpr2, Fcer1g, Ccr2, Ccr1, Cxcr4, C5ar1, Adra2a,* and *Adam8* (Figure 4B).

Simultaneously, construction of the PPI network was also established by STRING with a confidence score of ≥ 0.7 for significant differences. There were 1937 edges and 443 nodes in the PPI network (PPI enrichment *p*-value <0.001) (Figure 4C). Thirty MCODE modules were identified from the PPI network by the Cytoscape_MCODE. In particular, MCODE1 with the highest score comprised 24 genes: *Serpinb6b, Sirpb1c, Pirb, Aldh3b1, Sell, Lilrb4, C5ar1, Fabp5, Pira2, Mme, Tlr2, Siglece, Lgals3, Sirpb1b, H2-Bl, Fcgr4, Sirpb1a, Gp49a, Ticam2, Gm5150, Gm9733, Fpr1, Gm14548,* and *Serpinb10* (Figure 4D). With degree ≥ 10 considered as the standard of judgment, 10 genes were identified as hub genes with Cytoscape_cytoHubba: *Fpr2, Cxcl2, Fpr1, Cxcl1, Tnf, C5ar1, Serpinb6b, Aldh3b1, Tlr2,* and *Itgam* (Figure 4E).

**FIGURE 5:**
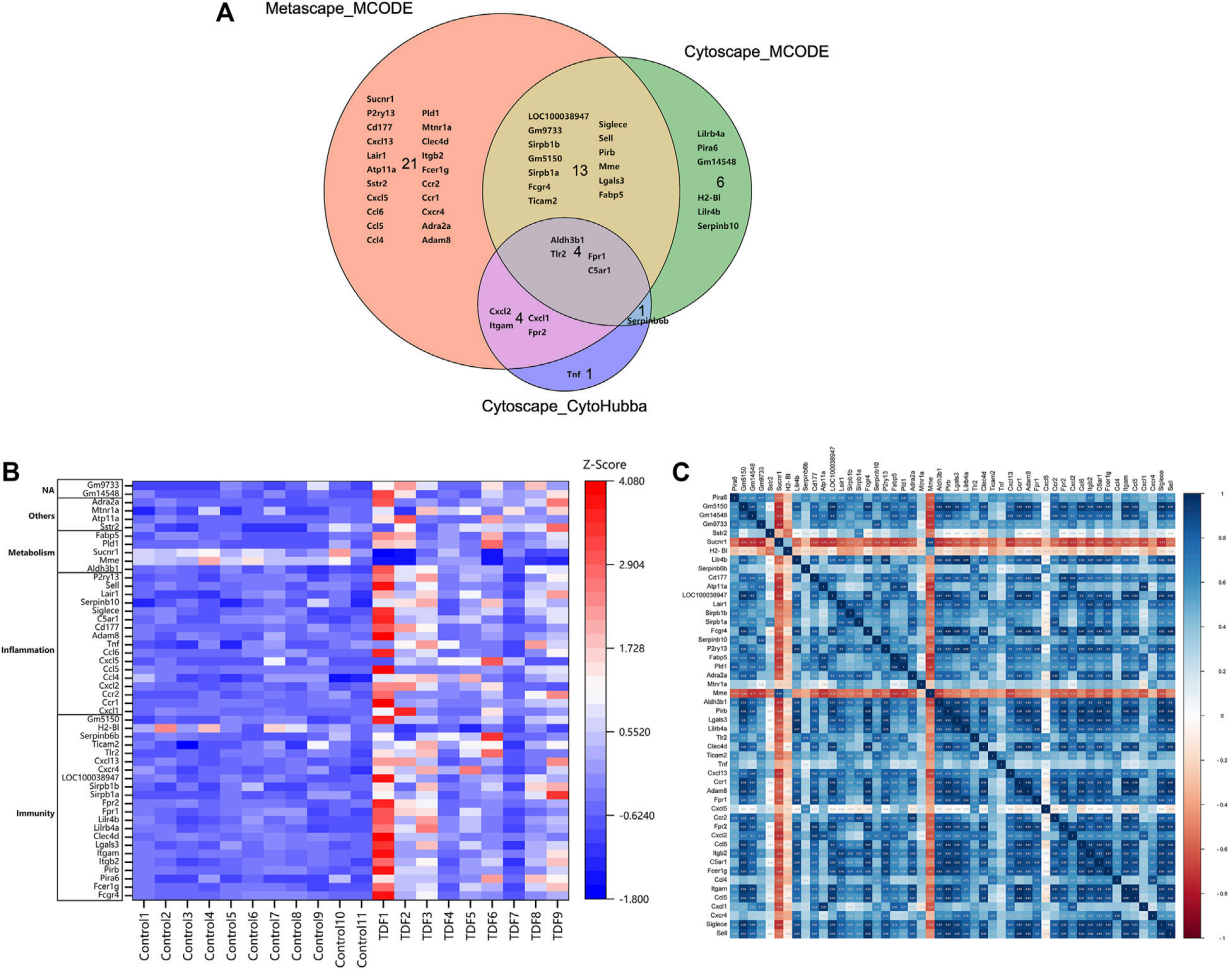
Identification of significant genes. **(A)** A VENN diagram of the 50 significant union genes among “Metascape_MCODE1,” “Cytoscape_MCODE1,” and “Cytoscape_cytoHubba.” **(B)** Hierarchical clustering heatmap and biological functional classification of the 50 significant genes. **(C)** Pearson correlation analysis of the 50 significant genes.

Finally, a VENN diagram was delineated and showed 50 significant union genes among “Metascape_MCODE1,” “Cytoscape_MCODE1,” and “Cytoscape_cytoHubba,” including four common genes: *Aldh3b1, Tlr2, Fpr1*, and *C5ar1* (Figure 5A). The basic information of the 50 significant genes is summarized in Supplementary Table S5. Hierarchical clustering indicated that the significant genes could largely differentiate the TDF group from the control group (Figure 5B). Moreover, we found that these 50 genes were involved mainly in “immunity” (44%, 22/50), “inflammation” (34%, 17/50), and “metabolic” processes (10%, 5/50), and six genes (12%, 6/50) exhibited other or undefined functions (Figure 5B). The Pearson correlation analysis showed a positive correlation among most genes except for *Sucnr1*, *H2-Bl*, *Mme*, and *Cxcl5* (Figure 5C).

### Nineteen Genes Were Identified to be the Potential Targets of TDF for Alleviating Nine CLDs Directly

**FIGURE 6:**
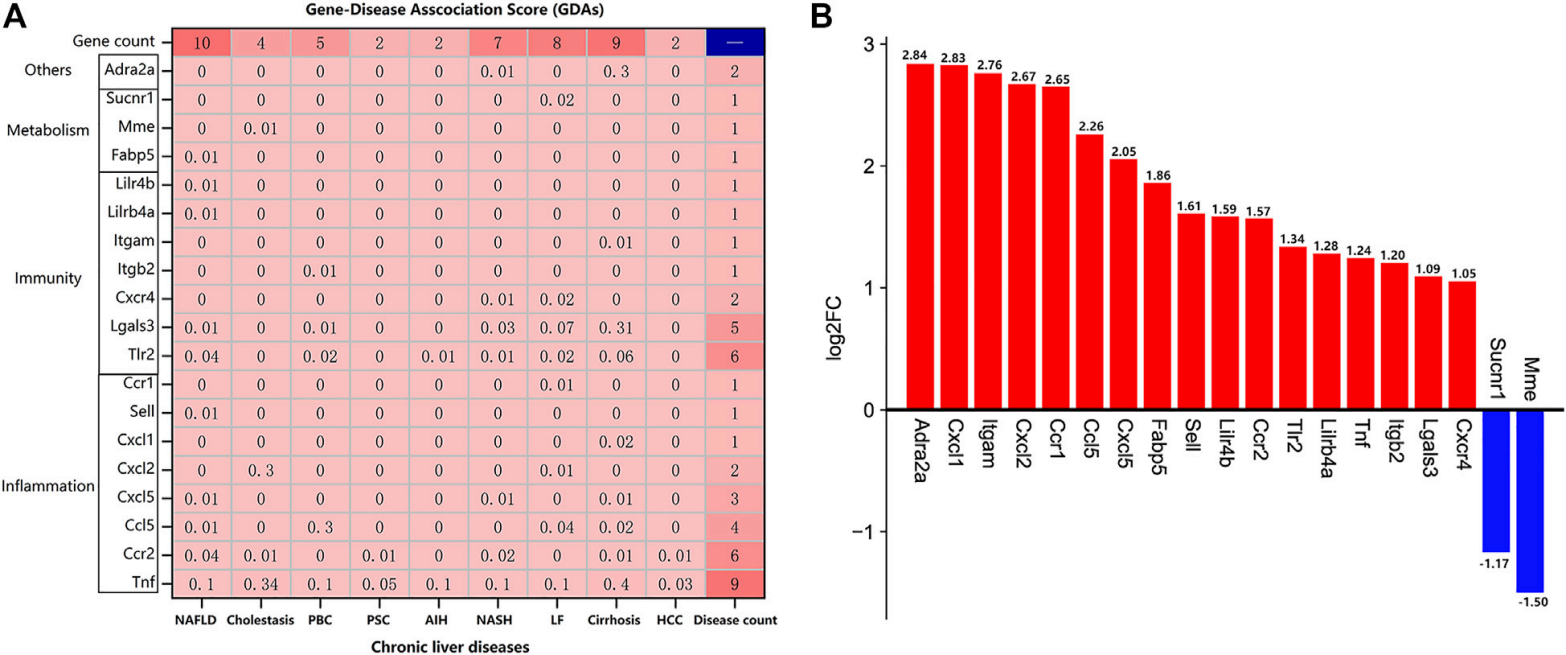
Identification of potential target genes of TDF for direct alleviation of chronic liver diseases (CLDs). **(A)** The gene–disease association (GDAs) score between the 19 potential target genes and nine CLDs. Gene count: the number of genes associated with a certain CLD; disease count: the number of CLDs associated with the corresponding gene. **(B)** The log2FC of the 19 potential target genes using RNA-seq data.

The DisGeNET platform was employed to identify the potential targets of TDF for alleviating CLDs. The nine most common CLDs including non-alcoholic fatty liver disease (NAFLD), cholestasis, primary biliary cholangitis (PBC), primary sclerosing cholangitis (PSC), autoimmune hepatitis (AIH), non-alcoholic steatohepatitis (NASH), liver fibrosis (LF), cirrhosis, and hepatocellular carcinoma (HCC) were analyzed. The gene–disease association score (GDAs) between the potential target genes and these CLDs are portrayed in Figure 6A. Nineteen genes were associated with these nine CLDs. The number of genes involved in “inflammatory,” “immune,” and “metabolic” processes was eight (42%), seven (37%), and three (16%), respectively. From the vertical perspective, 10 genes were related to NAFLD: *Tnf, Ccr2, Tlr2, Lgals3, Ccl5*, *Cxcl, Sell, Lilrb4a, Lilr4b,* and *Fabp5*. The four genes relevant to cholestasis were *Tnf, Ccr2, Cxcl2*, and *Mme*. Five genes (*Tnf, Tlr2, Lgals3, Ccl5*, and *Itgb2*) were connected with PBC. In addition, *Tnf* and *Ccr2* were associated with PSC. *Tnf* along with *Tlr2* were related to AIH. Moreover, seven genes were involved in NASH: *Tnf*, *Ccr2*, *Tlr2*, *Lgals3*, *Cxcl5*, *Cxcr4,* and *Adra2a*. Eight genes (*Tnf*, *Tlr2*, *Lgals3*, *Ccl5*, *Cxcl2*, *Cxcr4*, *Ccr1*, and *Sucnr1*) were relevant to LF. Besides, there were nine genes associated with cirrhosis: *Tnf, Ccr2, Tlr2, Lgals3, Ccl5, Cxcl5, Adra2a, Itgam*, and *Cxcl1*. *Tnf* together with *Ccr2* were related to HCC. In addition, according to the GDA score, the gene most associated with PBC was chemokine C-C motif ligand 5 (*Ccl5*), and the gene most closely related to the other eight liver diseases was *Tnf*. From the lateral perspective, we found that tumor necrosis factor (*Tnf*) was related to all of the nine CLDs, with cirrhosis (GDA score: 0.4) and cholestasis (GDA score: 0.34) showing the closest associations. Chemokine C-C motif receptor 2 (*Ccr2*) and toll-like receptor 2 (*Tlr2*) were associated with six CLDs, and galectin 3 (*Lgals3*) was related to five CLDs. The log_2_FC of genes indicated that 19 genes consisted of 17 upregulated DEGs and two downregulated DEGs, and the most significantly altered genes were adrenergic receptor alpha 2a (*Adra2a*), chemokine C-X-C motif ligand 1 (*Cxcl1*), and integrin alpha M (*Itgam*), with log_2_FC values of 2.84, 2.83, and 2.76, respectively (Figure 6B). The basic information of these genes can be found in Supplementary Table S5.

### A Total of 34 microRNAs (miRNAs) and 12 Transcription Factors (TFs) Were Predicted to be Upstream of the Nine Potential Target Genes

First, TF–gene analyses were undertaken with TRRUST, and 13 upstream TFs targeting 12 target genes (seven genes had no results) were identified (Table 1). Quite specifically, NF-κB family (*Nfkb1*, *Rela*, and *Rel*) were the most significantly enriched TFs and targeted seven genes (*Ccl5*, *Cxcl1*, *Cxcl2*, *Itgam*, *Tlr2*, *Tnf*, and *Cxcr4*). Additionally, the RNA-seq data showed that TDF could also affect expression of *Nfkb1* (log_2_FC = 0.22, Q-value <0.001), *Rela* (log_2_FC = 0.33, Q-value <0.001), and *Rel* (log_2_FC = 0.23, Q-value = 0.29). Overall, these results indicated that NF-κB might be the most critical TF for the genes targeted by TDF to relieve CLDs.

**TABLE 1:** Prediction of the transcription factors of target genes *via* TRRUST.

Key TF	Description	Overlapped genes	*p* Value	Q-value	List of overlapped genes	Log_2_FC (Q-value)
Nfkb1	Nuclear factor of kappa light polypeptide gene enhancer in B cells 1, p105	6	2.04E-08	2.65E-07	Ccl5, Cxcl1, Cxcl2, Itgam, Tnf, Tlr2	0.22 (<0.001)
Ikbkb	Inhibitor of kappaB kinase beta	3	1.51E-07	9.82E-07	Cxcl2, Tnf, Cxcr4	−0.14 (<0.001)
Irf1	Interferon regulatory factor 1	3	1.57E-06	6.79E-06	Ccl5, Tnf, Sell	−0.04 (0.21)
Rela	v-rel reticuloendotheliosis viral oncogene homolog A (avian)	4	4.30E-06	1.40E-05	Ccl5, Cxcr4, Tlr2, Tnf	0.33 (<0.001)
Jun	Jun proto-oncogene	4	5.90E-06	1.53E-05	Ccl5, Cxcl1, Cxcl2, Tnf	0.83 (<0.001)
Ar	Androgen receptor	2	6.41E-05	0.000139	Tnf, Ccr2	−0.30 (<0.001)
Irf8	Interferon regulatory factor 8	2	0.000207	0.000384	Tnf, Ccl5	0.39 (<0.001)
Rel	Reticuloendotheliosis oncogene	2	0.000308	0.000477	Ccl5, Tnf	0.23 (0.29)
Twist1	Twist basic helix-loop-helix transcription factor 1	2	0.000331	0.000477	Tnf, Mme	0.39 (0.06)
Spi1	Spleen focus forming virus (SFFV) proviral integration oncogene	2	0.000631	0.00082	Ccl5, Tnf	0.85 (<0.001)
Foxo1	Forkhead box O1	2	0.000947	0.00112	Sell, Ccr2	−0.26 (<0.001)
Sp1	*trans*-acting transcription factor 1	3	0.00191	0.00198	Sell, Tlr2, Tnf	0.04 (0.22)
Egr1	Early growth response 1	2	0.00198	0.00198	Tnf, Cxcl2	1.75 (<0.001)

Subsequently, DIANA-TarBase v8 was used to predict the upstream miRNAs of the 19 target genes, and 80 unique miRNAs were identified (Supplementary Table S6A). The most pivotal miRNAs are shown in Table 2. These results suggested that mmu-miR-155-5p was the most important miRNA and regulated 15 target genes.

**TABLE 2:** Prediction of the miRNAs of target genes *via* DIANA-TarBase.

miRNA name	Overlapped genes	List of overlapped genes
mmu-miR-155-5p	15	Cxcl1, Cxcl2, Cxcl5, Cxcr4, Ccl5, Ccr1, Ccr2, Sell, Lgals3, Tlr2, Fabp5, Tnf, Itgb2, Lilr4b, Lilrb4a
mmu-miR-1a-3p	9	Cxcl1, Cxcl5, Cxcr4, Ccl5, Ccr1, Itgb2, Tlr2, Fabp5, Adra2a
mmu-miR-21a-5p	8	Cxcl1, Cxcl2, Cxcl5, Ccr1, Sell, Tlr2, Cxcr4, Tnf
mmu-miR-122-5p	7	Cxcl1, Cxcr4, Ccl5, Ccr1, Tlr2, Sucnr1, Itgb2
mmu-miR-124-3p	6	Cxcl1, Cxcl5, Ccl5, Tlr2, Fabp5, Itgb2
mmu-miR-125b-5p	6	Ccl5, Ccr2, Sell, Adra2a, Cxcr4, Lilrb4a
mmu-miR-223-3p	5	Itgam, Fabp5, Sucnr1, Tnf, Lilr4b
mmu-miR-188-5p	4	Cxcl5, Ccl5, Tlr2, Mme
mmu-miR-196b-5p	4	Ccr2, Lgals3, Tnf, Lilr4b
mmu-let-7g-5p	3	Cxcl1, Cxcl5, Itgb2
mmu-let-7c-5p	3	Itgb2, Lgals3, Adra2a

Meanwhile, TF–miRNA interactions were predicted for the 13 identified TFs by using DIANA-TarBase, and 156 unique miRNAs were identified (Table S6B). Table 3 shows the most pivotal miRNAs. The results indicated that mmu-miR-155-5p and mmu-miR-124-3p, interacting with eight TFs, might be the most important miRNAs.

**TABLE 3:** Prediction of the miRNAs of identified transcription factors *via* DIANA-TarBase.

miRNA name	Overlapped TFs	List of overlapped TFs
mmu-miR-155-5p	8	Nfkb1, Jun, Ar, Irf8, Twist1, Spi1, Foxo1, Sp1
mmu-miR-124-3p	8	Nfkb1, Rela, Jun, Rel, Twist1, Foxo1, Sp1, Egr1
mmu-miR-106a-5p	6	Nfkb1, Irf1, Ar, Foxo1, Sp1, Egr1
mmu-miR-17-5p	6	Nfkb1, Irf1, Ar, Foxo1, Sp1, Egr1
mmu-miR-20b-5p	6	Nfkb1, Irf1, Ar, Foxo1, Sp1, Egr1
mmu-miR-1a-3p	6	Nfkb1, Jun, Rel, Twist1, Foxo1, Sp1
mmu-miR-93-5p	5	Nfkb1, Irf1, Foxo1, Sp1, Egr1
mmu-miR-22-3p	5	Nfkb1, Ikbkb, Ar, Foxo1, Sp1
mmu-miR-122-5p	5	Nfkb1, Rela, Jun, Ar, Foxo1
mmu-miR-125b-5p	5	Irf1, Jun, Irf8, Sp1, Egr1
mmu-miR-188-5p	5	Irf1, Rela, Ar, Twist1, Egr1
mmu-miR-26a-5p	5	Rela, Jun, Foxo1, Sp1, Egr1

**FIGURE 7:**
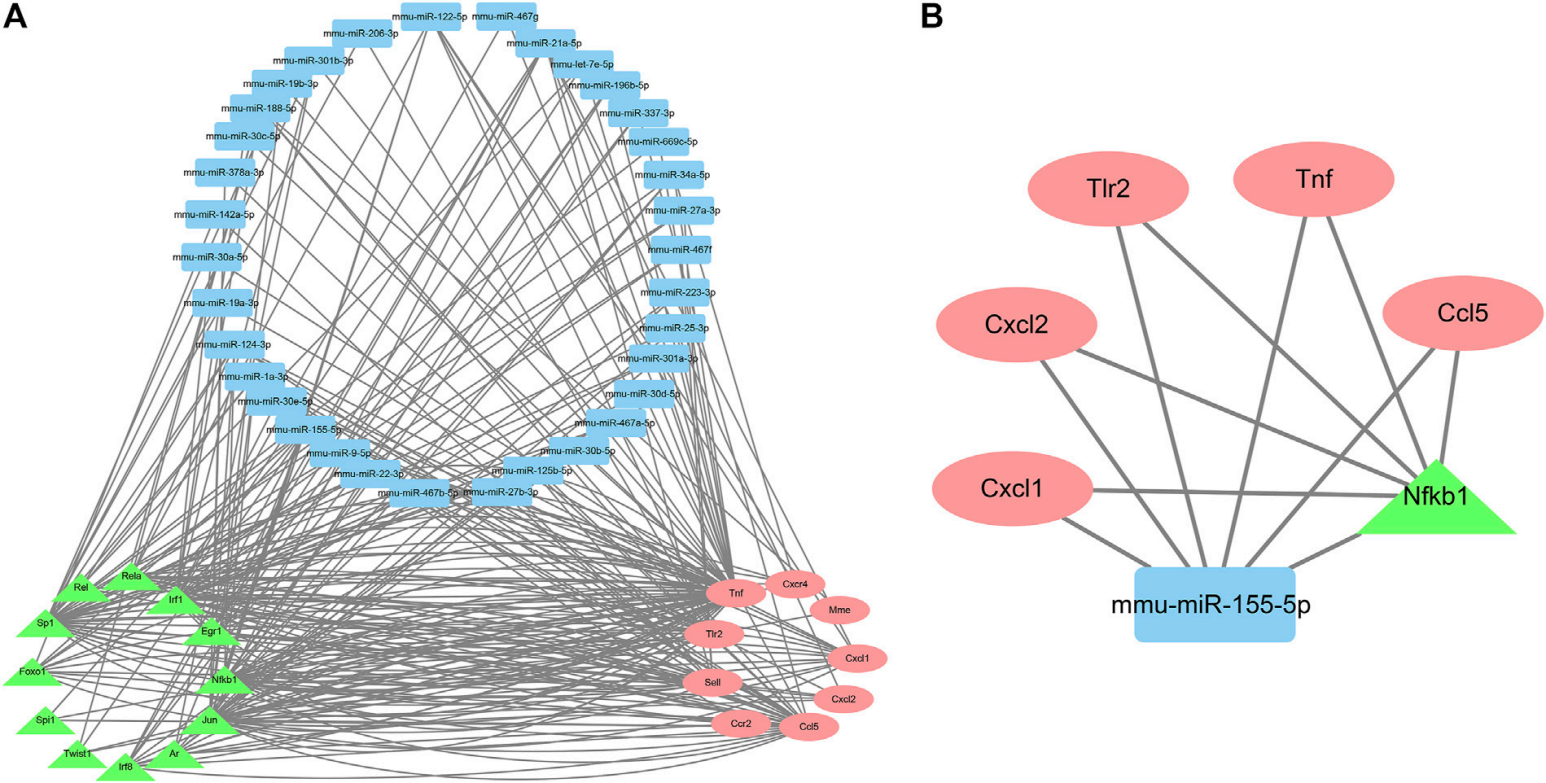
The TF-miRNA co-regulatory networks of the target genes. A. Co-regulatory networks consisting of nine genes, 12 TFs, and 34 miRNAs with pairwise relationships. B. Co-regulatory networks consisting of mmu-miR-155-5p and Nfkb1.

Finally, nine genes, 12 TFs, and 34 miRNAs that were pairwise-related were screened out to construct the TF–miRNA co-regulatory networks of the target genes, which consisted of 247 edges and 55 nodes (Figure 7A). These results indicated that mmu-miR-155-5p (involved in 15 edges) might be the most important miRNA. The most important TF might be Nfkb1 (19 edges). Their target genes were *Tnf*, *Ccl5*, *Tlr2*, *Cxcl1,* and *Cxcl2* (Figure 7B).

Taken together, these results suggested that co-regulatory networks consisting of mmu-miR-155-5p and Nfkb1 might be (at least in part) the underlying mechanisms by which TDF improved CLDs.

## Discussion

TDF has been recommended as a first-line oral antiviral agents by the European Association for the Study of the Liver (Lampertico et al., 2017) and American Association for the Study of Liver Diseases (Terrault et al., 2018) for treatment of chronic hepatitis B (CHB) patients due to its high efficacy and genetic barrier. Evidence suggests that CHB patients predispose towards other hepatic comorbidities (Liu et al., 2018; Oh et al., 2020), in addition to viral factors, in which immune, inflammatory, and metabolic disorders also have pivotal roles (Seto et al., 2018). Meanwhile, these chronic liver diseases (CLDs) can in turn affect the disease progression (Yu et al., 2017; Choi et al., 2020; Guo et al., 2020; Bockmann et al., 2021) and the efficacy of antiviral strategies in CHB patients (Jin et al., 2012; Kim et al., 2019). Consequently, we hypothesized that TDF can inhibit the replication of HBV (a major cause of CLDs) and also attenuate CLDs directly by regulating the hepatic immune, inflammatory, and metabolic status of the host. If this is true, then TDF could be a promising antiviral drug for CHB patients with other hepatic comorbidities.

In the current study, RNA-seq of murine livers from the TDF group and control group was carried out and 854 DEGs were identified (Figure 2). Analyses of functional enrichment showed that the DEGs were involved mainly in “immunity,” “inflammation,” and “metabolism” processes (Figure 3). Subsequently, 50 genes were screened out as significant genes by PPI construction and module analyses, and participated mainly in “immunity” (44%, 22/50), “inflammation” (34%, 17/50), and “metabolic” processes (10%, 5/50) (Figures 4, 5). Finally, 19 out of the 50 significant genes were identified to be potential target genes of TDF in alleviating nine CLDs directly, and were enriched in a greater proportion of “immunity” (37%, 7/19), “inflammation” (42%, 8/19), and “metabolic” processes (16%, 3/19) (Figure 6). Compelling studies have described immunity, inflammation, and metabolism to be closely related in the pathogenesis and progression of CLDs (Marra and Tacke, 2014; Heymann and Tacke, 2016; Tang et al., 2020; Ahmed et al., 2021). In addition, treatment strategies that synergistically affect hepatic immune, inflammatory, and metabolic states can regress CLDs (Hegade et al., 2016; Chen et al., 2019). Given that TDF could affect hepatic immune, inflammatory, and metabolic processes directly, one can speculate that TDF had the potential to alleviate CLDs independently of its antiviral activity.

Furthermore, it is well known that transcription factors (TFs) and microRNA (miRNAs) can jointly regulate target gene expression and contribute to multiple biological processes and different diseases. Notably, the mmu-miR-155-5p-NF-κB signaling pathway may have critical roles in CLDs by regulating expression of the genes involved in immune, inflammatory, and metabolic processes (Wang et al., 2009; Bala et al., 2011; Yuan et al., 2016; Qian et al., 2019; Ali et al., 2021). In this study, we predicted the upstream TFs and miRNAs of the 19 target genes. Then we screened nine genes, 12 TFs, and 34 miRNAs with pairwise relationships to construct the TF–miRNA co-regulatory networks of the target genes (Figure 7). mmu-miR-155-5p and Nfkb1 were identified to be the most important upstream moieties of these target genes. Hence, TDF might be able to attenuate CLDs by affecting hepatic immune, inflammatory, and metabolic processes by mmu-miR-155-5p-NF-κB signaling.

Our study had three main limitations. First, the enrichment analyses (using GO and KEGG databases) used in our study are based on the theory of over-representation analysis (ORA). This method considers only the DEGs list regardless of the expression and change in trends of DEGs, which may cause bias to some extent. However, considering that our study was qualitative, ORA is sufficient. Of course, the gene set enrichment analysis (GSEA) would be recommended for further studies. Second, only the direct effects of TDF on the liver were studied; other nucleoside/nucleotide analogs were not investigated. Hence, whether the direct ameliorative potential of TDF upon CLDs was unique to TDF or shared by all nucleoside/nucleotide analogs is not known. Future studies on ETV are needed to confirm this question. Third, the conclusions of our study are mainly from observations in immunocompetent mice with normal liver function, so inevitably the results will be a little overstated. Despite its descriptive nature, this study provides preliminary evidence that TDF affect the expression of genes associated with CLDs. Of course, we must verify the target genes and miRNAs by building corresponding disease models through *in vitro* and *in vivo* experiments. If we want to further translate our results to humans, chimeric mice with humanized liver is recommended to help us clarify the mechanism by which TDF improves a specific liver disease.

In conclusion, we report, for the first time, the hepatic transcriptional changes induced by TDF in healthy mice. Our findings indicate that TDF could ameliorate CLDs independently of its antiviral activity by influencing expression of the genes involved in hepatic immune, inflammatory, and metabolic processes *via* mmu-miR-155-5p-NF-κB signaling.

## Data Availability

The datasets presented in this study can be found in online repositories. The names of the repository/repositories and accession number(s) can be found below: https://www.ncbi.nlm.nih.gov/ PRJNA763152.
