# Supplementary material for: Potential Molecular Targets of Tenofovir Disoproxil Fumarate for Alleviating Chronic Liver Diseases via a Non-Antiviral Effect in a Normal Mouse Model
Source: Front Mol Biosci. 2021 Nov 16;8:763150. doi: 10.3389/fmolb.2021.763150 (PMC8635150; doi:10.3389/fmolb.2021.763150)
Supplement: Supplementary file 3 [file Table1.DOCX]

Supplementary Table 1A Summary of sequencing data filtering

| Sample | Raw Reads (M) | Clean Reads (M) | Clean Bases (Gb) | Clean Reads Q20 (%) | Clean Reads Q30 (%) | Clean Reads Ratio (%) |
| --- | --- | --- | --- | --- | --- | --- |
| Control1 | 23.78 | 23.39 | 1.17 | 98.54 | 94.08 | 98.37 |
| Control2 | 23.78 | 23.39 | 1.17 | 98.54 | 94.19 | 98.33 |
| Control3 | 23.78 | 23.39 | 1.17 | 98.47 | 93.95 | 98.34 |
| Control4 | 23.78 | 23.4 | 1.17 | 98.56 | 94.18 | 98.38 |
| Control5 | 23.78 | 23.44 | 1.17 | 98.6 | 94.6 | 98.55 |
| Control6 | 23.78 | 23.35 | 1.17 | 98.54 | 94.18 | 98.18 |
| Control7 | 23.67 | 22.63 | 1.13 | 98.46 | 93.81 | 95.62 |
| Control8 | 23.69 | 22.73 | 1.14 | 98.52 | 94.13 | 95.93 |
| Control9 | 23.68 | 22.67 | 1.13 | 98.46 | 93.86 | 95.71 |
| Control10 | 23.68 | 22.67 | 1.13 | 98.48 | 93.78 | 95.73 |
| Control11 | 23.67 | 22.64 | 1.13 | 98.51 | 94 | 95.64 |
| TDF1 | 23.67 | 22.63 | 1.13 | 98.45 | 93.69 | 95.62 |
| TDF2 | 23.68 | 22.69 | 1.13 | 98.5 | 93.85 | 95.83 |
| TDF3 | 23.69 | 22.74 | 1.14 | 98.46 | 93.58 | 95.98 |
| TDF4 | 23.7 | 22.77 | 1.14 | 98.53 | 94.05 | 96.07 |
| TDF5 | 23.69 | 22.72 | 1.14 | 98.48 | 93.93 | 95.94 |
| TDF6 | 23.67 | 22.63 | 1.13 | 98.4 | 93.36 | 95.62 |
| TDF7 | 23.65 | 22.56 | 1.13 | 98.43 | 93.87 | 95.39 |
| TDF8 | 23.68 | 22.66 | 1.13 | 98.52 | 94.26 | 95.71 |
| TDF9 | 23.74 | 23.11 | 1.16 | 98.61 | 94.19 | 97.36 |

M: Million; Gb: giga-base; Q20/30: The percentage of bases with a quality value≥20/30.

Supplementary Table 1B Summary of clean reads mapped to the reference genome

| Sample | Total Clean Reads (M) | Total Mapping (%) | Uniquely Mapping (%) |
| --- | --- | --- | --- |
| Control1 | 23.39 | 96.69 | 77.74 |
| Control2 | 23.39 | 96.89 | 78.09 |
| Control3 | 23.39 | 96.64 | 77.85 |
| Control4 | 23.4 | 96.83 | 77.31 |
| Control5 | 23.44 | 96.89 | 77.32 |
| Control6 | 23.35 | 96.67 | 76.32 |
| Control7 | 22.63 | 96.69 | 77.9 |
| Control8 | 22.73 | 96.67 | 77.43 |
| Control9 | 22.67 | 96.73 | 79.8 |
| Control10 | 22.67 | 96.93 | 78.49 |
| Control11 | 22.64 | 96.86 | 78.94 |
| TDF1 | 22.63 | 95.75 | 78.83 |
| TDF2 | 22.69 | 96.92 | 81.35 |
| TDF3 | 22.74 | 96.72 | 78.63 |
| TDF4 | 22.77 | 96.96 | 78.13 |
| TDF5 | 22.72 | 96.91 | 79.44 |
| TDF6 | 22.63 | 97.01 | 81.02 |
| TDF7 | 22.56 | 96.92 | 78.44 |
| TDF8 | 22.66 | 96.9 | 79.59 |
| TDF9 | 23.11 | 97.04 | 80.59 |

M: Million
