# Supplementary material for: Potential Molecular Targets of Tenofovir Disoproxil Fumarate for Alleviating Chronic Liver Diseases via a Non-Antiviral Effect in a Normal Mouse Model
Source: Front Mol Biosci. 2021 Nov 16;8:763150. doi: 10.3389/fmolb.2021.763150 (PMC8635150; doi:10.3389/fmolb.2021.763150)
Supplement: Supplementary file 5 [file DataSheet1.PDF]

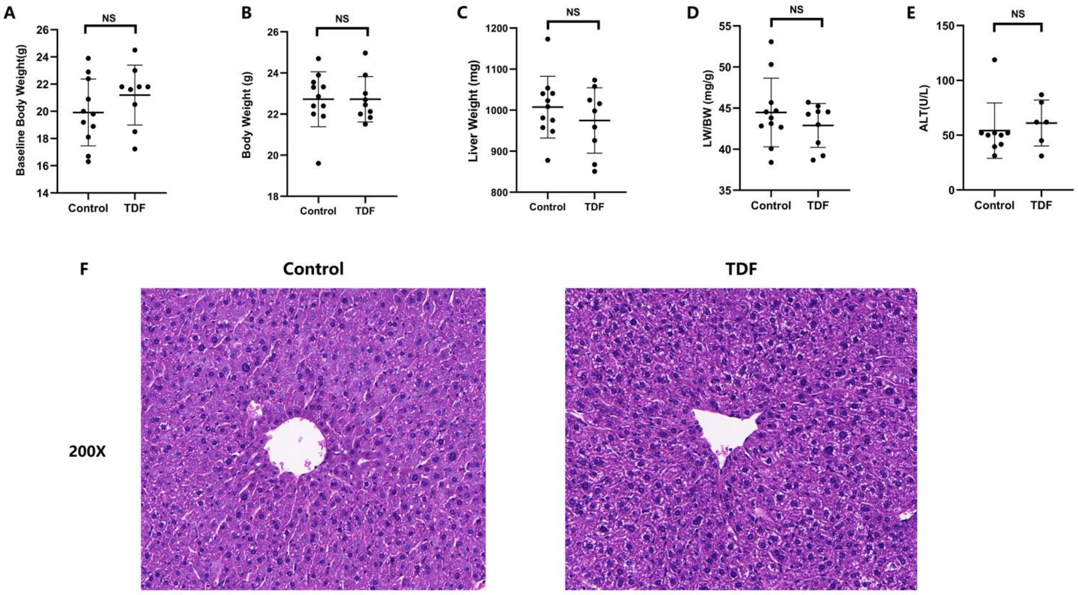

Supplementary Figure 1. TDF had no significant effect on liver function of normal mice. (A) Body weight of mice in control group (n=11) and TDF group (n=9) at baseline. Body weight (BW, B), Liver weight (LW, C), liver index (LW/BW, D) of mice in control group (n=11) and TDF group (n=9) at the end of the experiment. (E) ALT level of mice in control group (n=9) and TDF group (n=6) at study termination. (F) Liver tissues were stained with hematoxylin–eosin (200x). NS: No Significant.
